# A Review of Photonic Sensors Based on Ring Resonator Structures: Three Widely Used Platforms and Implications of Sensing Applications

**DOI:** 10.3390/mi14051080

**Published:** 2023-05-20

**Authors:** Nikolay L. Kazanskiy, Svetlana N. Khonina, Muhammad A. Butt

**Affiliations:** 1 Department of Technical Cybernetics, Samara National Research University, 443086 Samara, Russia; 2 IPSI RAS—Branch of the FSRC “Crystallography and Photonics” RAS, 443001 Samara, Russia

**Keywords:** ring resonator, plasmonics, semiconductor, silicon-on-insulator, polymers, sensors, biosensing, temperature sensing, gas sensing

## Abstract

Optical ring resonators (RRs) are a novel sensing device that has recently been developed for several sensing applications. In this review, RR structures based on three widely explored platforms, namely silicon-on-insulator (SOI), polymers, and plasmonics, are reviewed. The adaptability of these platforms allows for compatibility with different fabrication processes and integration with other photonic components, providing flexibility in designing and implementing various photonic devices and systems. Optical RRs are typically small, making them suitable for integration into compact photonic circuits. Their compactness allows for high device density and integration with other optical components, enabling complex and multifunctional photonic systems. RR devices realized on the plasmonic platform are highly attractive, as they offer extremely high sensitivity and a small footprint. However, the biggest challenge to overcome is the high fabrication demand related to such nanoscale devices, which limits their commercialization.

## 1. Introduction

Even though optical waveguides (WGs) were initially designed for use in the telecommunications industry, it was quickly discovered that their mechanical stability, flexible geometry, noise immunity, and effective light-conducting over long distances make them well-suited for use in sensor applications. Due to their affordability, compact size, and flexible geometry, fiber optics have formed the foundation of the most advanced optical WG sensors to date. However, since they must accommodate the telecoms market’s offerings, optical fibers cannot be created for one specific purpose. Because of this, in recent years, the development of planar or channel optical WGs for sensors has received more attention.

A ring resonator (RR) sensor is a type of optical sensor that is based on the principle of resonant light coupling in a ring-shaped WG [1]. This sensor typically consists of a ring-shaped WG that is made from a high-refractive-index material, such as silicon (Si), and is coupled with a bus WG [2]. The RR is designed to have a specific resonant frequency that is determined by its dimensions and refractive index (RI). When light is coupled into the WG, it circulates in the ring and interferes with itself [3]. The resonant frequency of the ring corresponds to the frequency at which the light waves constructively interfere, resulting in a large signal that can be detected by an external photodetector [4]. Any changes in the RI of the surrounding medium can affect the resonant frequency of the ring, causing a shift in the output signal. This shift can be used to measure the concentration or presence of analytes in the surrounding medium [5]. RR sensors have many applications in various areas, for example biomedical sensing, environmental monitoring, and chemical analysis. They offer high sensitivity, low detection limits, and label-free detection, making them desirable for a wide range of sensing functions [6,7].

An RR’s use as a standalone device depends on its connectivity to the outside environment. Codirectional evanescent coupling between the ring and a nearby bus WG is the most typical coupling method. A single RR in a bus WG will cause dips in the transmission spectrum near the ring resonances. In this manner, the RR operates as a spectrum filter that may be applied to optical communication, particularly wavelength division multiplexing (WDM). The position and form of the resonance dips are extremely sensitive to some influences, which can be useful (as a sensor, or for tuning) or disadvantageous (as a filter’s stability). As an alternative, these ring spectra can be employed for sensing. 

RRs are useful components that can be employed in a wide range of applications in photonics and telecommunications. A few examples are as follows: they can be utilized as narrowband filters in optical communication systems. They can be used to select or filter out specific wavelengths of light, which is useful in applications such as WDM and optical add-drop multiplexing (OADM) [8]. RRs can be used as optical modulators. By altering the resonant wavelength of the RR, the intensity of the transmitted light can be modulated. This can be of use in applications such as optical data transmission [9]. RRs can be applied as sensors for detecting variations in the RI of the surrounding medium. This can be useful in applications such as chemical, temperature, pressure, and biological sensing. RRs can be used in nonlinear optics, where the intensity of the light induces nonlinear effects in the material of the resonator. This can be used for applications such as frequency doubling and the generation of entangled photons. RRs can be used as components in quantum computing systems, where they can be used to generate and manipulate entangled photons [10].

Semiconductor materials, such as Si, or compound semiconductors such as indium phosphide (InP) or gallium arsenide (GaAs), offer several advantages for integrated optics due to their unique properties [11]. Semiconductor materials typically have higher refractive indices compared to other materials, such as polymers or oxides [12]. This high RI contrast is advantageous for the design and fabrication of compact photonic components, including WGs, couplers, and resonators [13]. It enables efficient light confinement and low-loss propagation, resulting in highly integrated and miniaturized optical circuits [14]. Si is especially notable as a semiconductor platform for integrated optics due to its compatibility with complementary metal–oxide–semiconductor (CMOS) processes [15]. CMOS technology is widely used in the semiconductor industry for fabricating electronic circuits, and leveraging the existing infrastructure allows for the cost-effective integration of optics and electronics on a single chip. Si photonics has emerged as a promising field, enabling the monolithic integration of high-speed optical devices and CMOS electronics [16].

Many polymers have excellent transparency in the visible spectrum, allowing light to pass through with minimal absorption or scattering. This property is crucial for optical devices as it enables efficient transmission of light without significant loss [17]. Polymers can be engineered to have high flexibility, making them suitable for applications where bending or shaping is required. This property is especially valuable in the production of optical fibers, WGs, or flexible displays, where the material needs to conform to specific shapes or be bent without losing its optical properties. Polymers are often more cost-effective compared to traditional optical materials such as glass or crystal [18]. They can be manufactured using scalable and relatively inexpensive processes, making them a viable option for mass production of optical devices [19,20,21]. 

**Figure 1:**
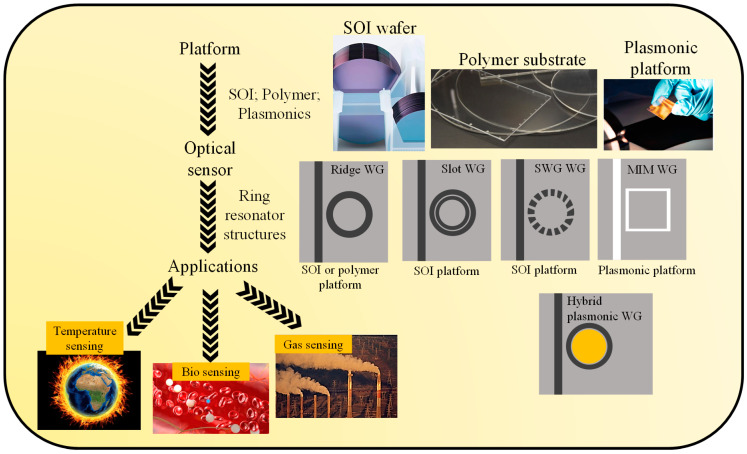
Optical ring resonators are based on different optical platforms such as SOI wafers [24], polymers [25], and plasmonics [26] for biosensing [30], temperature sensing [31], and gas sensing applications [32].

Plasmonic materials exhibit a phenomenon called surface plasmon resonance (SPR), where the collective oscillation of electrons is resonantly excited by incident light [22]. This resonance can be precisely tuned by adjusting the geometry, size, and material properties of the nanostructures, enabling control over the interaction of light with the material. SPR is widely used in biosensing applications, where changes in the RI near the metal surface can be detected as shifts in the resonance, allowing for highly sensitive detection of biomolecules or chemical species [23]. In this review, the most widely used photonic platforms such as semiconductors [24], polymers [25], and plasmonics [26] for the realization of RR-based devices are extensively studied, as shown in Figure 1. We aim to discuss the sensing capabilities of RRs developed on these attractive platforms and their sensing performances [27,28,29]. 

## 2. Working Principle of RR Structure

The working principle of an RR is based on the phenomenon of resonance. When light is injected into the RR, it travels around the loop (ring) and interferes with itself. The interference can either be constructive or destructive, depending on the wavelength of the light and the length of the loop. If the length of the loop is equal to an integer multiple of half the wavelength of the light, then the interference will be constructive, and the light will be trapped inside the loop. This is known as the resonance condition, which can be expressed as [1]:

2πrn_eff_ = mλ_res_, (1)

where r is the radius of the RR, λ_res_ is the wavelength of the light, m is an integer, n_eff_ is the effective index of the WG, and π is the mathematical constant pi. When the resonance condition is met, the light continues to circulate in the RR, and the intensity of the light builds up due to constructive interference. This effect can be used to enhance or filter specific wavelengths of light. By adjusting the radius of the RR, the resonance condition can be tuned to a specific wavelength. 

**Figure 2:**
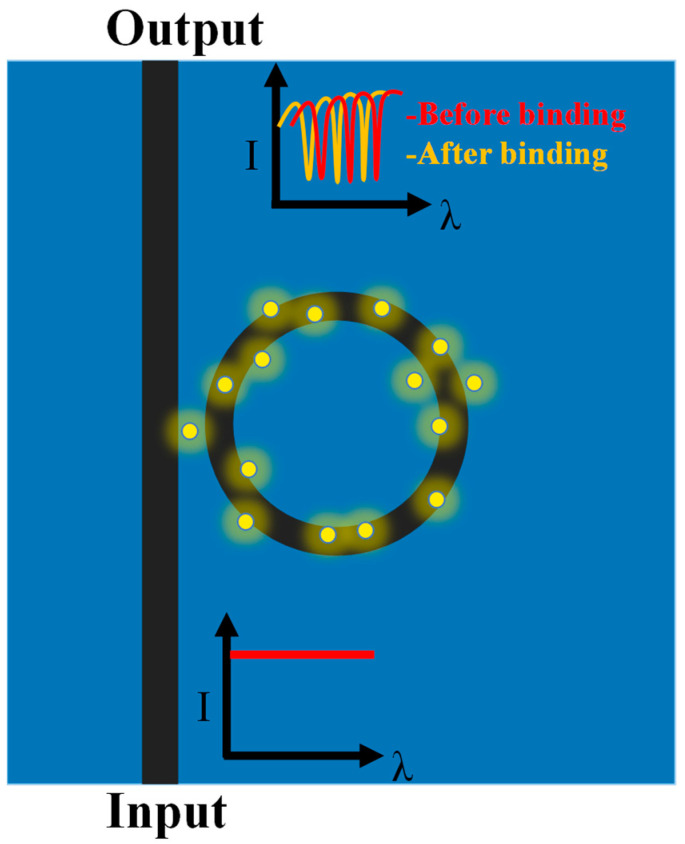
Resonance wavelength shift in the RR due to the change in the ambient RI.

Evanescent field sensing, however, underpins their overall mode of functioning. Resonator resonance conditions can change, causing a resonance wavelength shift, if the evanescent field is altered as a result of analytes becoming immobilized on the WG, as shown in Figure 2. The sensitivity of the RR device under the influence of change in the external RI/temperature/gas can be expressed as

(2) $$S\left(\frac{nm}{RIU}\right)=\frac{∆\lambda }{\Delta n}\;or\;S\left(\frac{nm}{o_{C}}\right)=\frac{∆\lambda }{∆T}\;or\;S(\frac{nm}{ppm})=\frac{∆\lambda }{∆conc.};$$

where ∆*λ* is the change in the resonance wavelength, whereas ∆*n*, ∆*T*, and ∆*conc*. are the change in ambient RI, temperature, and gas concentration, respectively. By detecting either the resonance peak shift or the intensity change, antibodies that only bind to their respective antigens are thereby recognized with excellent specificity. The residuals can be eliminated by drying or flushing after the analyte–antibody binding has occurred to improve the specific measurement.

A prerequisite for obtaining excellent performance is the suppression of technical temperature changes, as the resonator’s radius and refractive index fluctuate with temperature, which also affects the resonance frequencies of its modes [33,34]. To protect the mode volume from temperature and humidity instabilities, this often includes technically challenging procedures [35]. If the appropriate resonator temperature can be precisely determined and its variations are adjusted, the criteria for the shielding may be less strict [36]. To do this, making use of the differential frequency shift caused by a change in temperature between two orthogonally polarized whispering gallery modes (WGMs) in an optically anisotropic crystal resonator has been suggested [37]. This “dual-mode frequency” change can therefore act as a precise thermometer. This method, also known as the dual-mode technique, might potentially result in a “stability transformer” that enhances the stability of radio-frequency standards while also simplifying laser frequency stabilization methods [38,39].

The RR has a variety of unique features as novel sensing technologies. The physical length of the sensor is the length of a standard linear WG or fiber-based sensor, but the circular nature of the resonant mode results in an extraordinarily long effective interaction length, which is governed by

(3) $$L_{eff}=\frac{Q\lambda }{2\pi n};$$

where *Q* is the number of trips that circulating resonant light may take along the RR, often known as the *Q*-factor for resonators [40]. The *Q*-factor typically ranges from 10^4^ to 10^8^ according to the RR layout. Due to its effective contact length, which is many tens of centimeters or even longer despite its modest physical size, the RR offers improved sensing performance, a reduced environmental impact, and higher multiplexing capability while requiring less analyte. An additional benefit of the RR is the greatly increased light intensity near its surface, with the augmentation being proportional to the *Q*-factor. This is because, once more, the resonant light circulates, and this is why. Sensing applications can potentially make use of this phenomenon.

## 3. WG Structures versus Sensitivity versus Fabrication Complexity

Optical WGs are formations that are designed to confine and guide light along a specific path. There are several types of optical WG structures, such as rib, ridge, slot, and subwavelength grating (SWG), hybrid plasmonic, and purely plasmonic WGs, each with its unique properties and applications. In sensing applications, WGs are most frequently utilized in three main categories. These include ridge [41], rib [42], and slot WGs [43]. The evanescent field of the guided mode considerably penetrates the top cladding material, which holds the analyte. Each WG architecture has a different quantity of light entering the upper cladding, and this variance correlates with unfavorable optical losses; the more light entering the upper cladding, the greater the optical losses due to absorption and scattering. 

**Figure 3:**
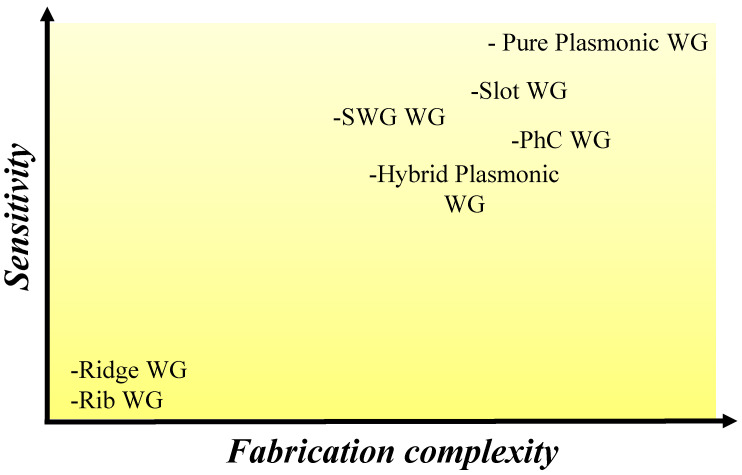
Different WG structures and their sensitivity and fabrication complexity comparison.

In WG architectures such as ridge and rib, light is primarily constrained inside the high index core [44,45]. However, in slot WG designs, light can be significantly trapped in the subwavelength low-index medium sandwiched between two high-index rails. Due to the greater spatial interplay between the sensing medium and evanescent field, slot WGs are significantly more sensitive than ridge WGs [46]. The use of slot WGs for bulk index sensing is therefore popular [47]. It is important to choose an appropriate WG geometry for the given situation. The sensitivity of ridge and rib WGs is sacrificed to achieve low optical losses. However, slot WGs have a significant optical loss despite having excellent sensitivity [7]. Figure 3 illustrates the relationship between the sensitivity and fabrication complications of different WG architectures. 

Other attractive WG architectures include SWG, PhC, hybrid plasmonic, and purely plasmonic WGs which offer high sensitivity and have been extensively researched in recent times for diverse applications [23,48,49,50]. Practically, the ridge and rib WGs can be fabricated via a standard CMOS fabrication process, which is a widely used technique for manufacturing photonic integrated circuits (PICs) such as microprocessors, memory chips, and other electronic components [51,52]. Conventional photolithography processes along with the reactive ion etching (RIE) method are suitable to obtain desirable resolution [53]. Conversely, SWG, PhC, and MIM WGs require rather high resolution, which can be obtained via electron beam lithography (EBL) [54]. One advantage of EBL is its high-resolution capability. The small wavelength of electrons allows for the creation of features as small as a few nanometers. This makes EBL suitable for fabricating nanostructures with high precision and complexity. However, EBL has some limitations as well. It is a relatively slow process compared to optical lithography, and large-area patterning can be time-consuming. Additionally, the cost of equipment and the difficulty of the process make it less practical for routine manufacturing applications.

## 4. RR Sensors Based on Different Platforms and Potential Applications

In this section, RR structures based on SOI, polymer, and plasmonic platforms are extensively discussed for temperature, gas, and biosensing applications. Additionally, polymers typically possess good elasticity, allowing them to deform under applied forces and return to their original shape when the forces are removed. This property enables polymer sensors to accurately measure strain or deformation. Therefore, a short discussion on polymer-based mechanical sensors is also presented. These photonic platforms have been widely explored, and several commercial devices are commercially available.

### 4.1. Silicon-on-Insulatorplatform

The supremacy of low-propagation-loss, high-RI, and CMOS-compatible manufacturing techniques is demonstrated by silicon-on-insulator (SOI) on-chip photonic devices [55]. These devices are regarded as the most viable technological platform for implementing photonics-integrated circuits (PICs) for optical interconnects, biosensing, and data processing [48]. In areas including fundamental medical research and diagnosis, smart home healthcare diagnostics, and gas sensing applications, Si photonic sensors have been extensively studied for usage as biosensors, temperature sensors, and gas sensors [56]. These sensors should have high sensitivity, which is strongly affected by the size of the device, optical loss, the polarization of the light, and the overlap between the light and the surrounding material. There is always a trade-off, because none of these performance measures can be maximized simultaneously [57]. Hence, it is necessary to develop such sensors that are economical and simple to manufacture based on currently existing technology. The optical confinement factor of covering or infiltrating materials has been improved by using a variety of WG architectures, such as hollow-core [58], subwavelength grating [46], slot [59,60], and plasmonic slot WGs [61]. This increases sensitivity.

Since Si provides RRs beyond a comparably small size, these structures are crucial to the development of Si photonics. An optical WG that has been looped back on itself forms the basis of a general RR, which reaches resonance when its optical path length equals exactly one wavelength. Therefore, RRs may sustain numerous resonances, and the free spectral range (FSR), which measures the distance between these resonances, is dependent on the resonator length. Many applications want a rather large FSR (a few nm), which necessitates the usage of tiny rings. This results in an extremely difficult condition for the optical WG: to form a compact ring, a tiny bend radius is needed, and only high-contrast WGs with strong confinement can do this.

On the SOI platform, the sensitivity of the μ-RR-based sensors is less than 100 nm/RIU. The use of subwavelength grating RR, however, enables the achievement of a sensitivity of 672.8 nm/RIU [62,63]. The SOI and III-V platforms’ capabilities are greatly increased via hybrid integration, enabling the construction of intricate sensor systems with active components [64,65]. The SOI platform’s capabilities can further be increased by utilizing IMOS (indium phosphide membrane on Si) technology [66].

Using a Si photonic temperature sensor with a cascaded ring resonator (CRR) to simultaneously increase sensitivity and range has been suggested [67]. The proposed CRR temperature sensor uses two μ-RRs with distinct temperature sensitivity and free spectral ranges (FSRs) to achieve dual enhancement. By customizing the in-plane geometric parameters of the two RRs, variations in the temperature sensitivity and FSRs are produced. A single-mask CMOS-compatible technique was used to create the CRR temperature sensor. The temperature sensitivity measured in the experiments was ~293.9 pm/°C, which was 6.3 times more than that of a single RR. It was also demonstrated that the sensor could increase the temperature detecting range by 5.3 times [67].

**Figure 4:**
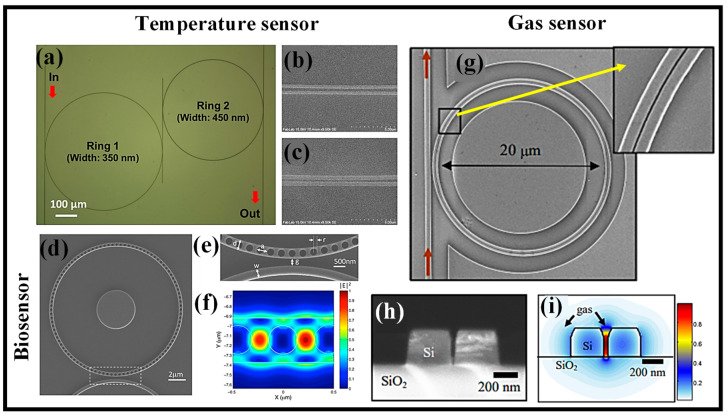
Semiconductor RRs for different applications, (**a**) Optical microscope image of the manufactured CRR temperature sensor [67], (**b**) SEM image of the ring and bus WG in the coupling region for ring 1 [67], (**c**) SEM image of the ring and the bus WG in the coupling region for ring 2 [67], (**d**) SEM image of the manufactured PhC μ-RR [72], (**e**) zoomed-in SEM image of the coupled region [72], (**f**) computational optical field distribution in the ring [72], (**g**) SEM image of the Si slotted μ-RR [75], (**h**) cross-sectional SEM image of a slot WG [75], (**i**) computer mode profile for E-field component of the fundamental quasi-TE mode [75].

A 210 nm-thick Si layer and a buried 3 μm-thick oxide layer were used to create the CRR temperature sensor. The ring and bus WGs were patterned using e-beam lithography after the SOI wafer had been spin-coated with poly(methyl methacrylate) (PMMA). On top of the patterned PMMA layer, a 20 nm-thick chromium (Cr) layer was applied next, and this was followed by the Cr lift-off procedure. Inductively coupled plasma reactive ion etching (ICP-RIE) was used to create the ring and bus WGs. The plasma-enhanced chemical vapor deposition (PECVD) method was used to create a 1 μm-thick oxide cladding layer after the Cr etch mask was removed. The optical microscopy image and SEM images of the manufactured sensor are shown in Figure 4a and Figure 4b,c, respectively [67].

To regulate the propagation of light, Yablonovitch and John created the artificial dielectric structure known as photonic crystals (PhCs) in 1987 [68]. PhCs have periodic and random variations in the RI. The capacity to precisely control the electromagnetic (EM) field’s propagation inside these structures may be used by the photonic devices created on PhCs. Additionally, it is possible to create devices with small footprints [48]. Recently, various intriguing devices based on PhCs have been proposed, including Y-branches, small-diameter bent WGs, and miniature resonator cavities [69,70]. These remarkable qualities may facilitate the creation of a dense integrated circuit [71].

A one-dimensional PhC μ-RR-based label-free optical biosensor with improved light–matter interaction is developed [72]. In comparison with traditional μ-RR sensors, a sensitivity improvement of more than two times is made in volumetric and surface sensing. Label-free detection of DNA and proteins at the nanomolar scale is described, and the experimental bulk detection sensitivity is 248 nm/RIU. The PhC μ-RR biosensor may be produced using the same common lithographic procedures as conventional μ-RRs with a minimum feature size greater than 100 nm. Figure 4d displays a top-view picture of the PhC RR taken using an SEM [72]. The structure has a device layer that is 220 nm thick and is constructed on an SOI platform. The Si layer is etched through to the buried oxide layer. On the RR, there are N = 100 circular air holes, giving the ring a 7.16 μm radius. Figure 4e depicts a top-view enlarged SEM picture of the device in the coupling regime [72]. An illustration of the PhC RR’s on-resonance optical mode field profile in an area of the PhC WG is shown in Figure 4f [72]. 

A key feature of sensors and applications involving isolated atomic or molecule states in quantum optics is the interaction of light with gaseous matter [73]. The bulk of room-temperature investigations on interactions between light and matter in integrated photonic devices have used solid or liquid matter [74]. This is mostly caused by the frequently negligible changes in RI and absorption between gases at optical wavelengths. Utilizing the improved optical interaction at atomic resonances, on-chip optical interaction with Rb vapor in ARROW WGs was recently demonstrated [73]. On-chip room-temperature optical interaction with gasses has not been studied in the absence of such atomic resonances because of the moderate strength of the interaction.

A high-confinement resonant cavity made from slotted WGs for on-chip optical interrogation of inadequately interacting gases was used [49,76]. This improves the light–matter interaction with the gas by using slot WG geometry in combination with resonant cavities, which have been shown to be incredibly effective as sensors of change in RI [1,49,77]. To show that this can detect even minor variations in the RI of surrounding gases, the improved light–matter interaction of the slot WG is combined with the RI sensitivity of the μ-RR. A slotted Si μ-RR is used in a chip-scale photonic system that can detect the composition and pressure of gases at ambient temperature. In the near-IR, swings in the resonance wavelength caused by the presence and pressure of acetylene gas are observed, along with variations in the RI that are as tiny as 10^−4^. The device’s actual sensitivity, which is improved by the slot-WG design, is in line with the predicted value of 490 nm/RIU [75]. Figure 4g depicts an SEM image of a Si μ-RR with a 20 μm diameter like that utilized for gas detection [75]. A cross-sectional SEM of a slot WG like the one employed in our device is shown in Figure 4h [75]. Based on this geometry, the basic quasi-TE mode is determined, and the main E-field component is shown in Figure 4i using a finite difference mode solver [75]. Table 1 presents recently demonstrated semiconductor-based RR devices for gas, temperature, and biosensing applications. 

### 4.2. Polymer Platform

Polymers offer numerous advantages for integrated optics compared to other materials, such as Si or glass. Polymers are relatively cheap to manufacture compared to traditional materials such as Si or lithium niobate. This cost–benefit makes polymer-based integrated optics more accessible for large-scale production and deployment. Polymer materials can be easily processed using techniques such as spin coating, photolithography, and imprinting, allowing for the fabrication of complex optical structures with high precision [85]. This flexibility in fabrication enables the design and production of customized and application-specific integrated optical devices. 

In the past few years, several researchers have examined the creation of optical WGs employing hot embossing [18,86,87,88]. Many stamp production techniques were investigated, including LIGA technology, photolithography, micro-machining, and etching. Thermoplastics and photoresists were also examined as part of the examination of various polymer materials [89]. The hot embossing procedure, while having its benefits, also has certain drawbacks, such as the need for specific tools, which can be expensive and may not be accessible to all users. High temperatures are needed for the hot embossing process, which can lead to thermal stress and the breakdown of the polymer substance. It needs intricate tooling, such as molds and stamps, which can be challenging to design and make. Additionally, it is restricted to specific types of thermoplastic polymer materials, which might not be appropriate for all applications. The embossed component’s surface polish may not be as good as that of other production techniques, which might have an impact on the component’s optical characteristics and lead to uneven embossing that could lower the component’s performance. In addition, because it is a labor-intensive and generally slow process, it might not be appropriate for high-volume production.

Polymers have low density, making them lightweight compared to other materials. This characteristic is particularly advantageous for applications where weight and size constraints are important, such as in portable or wearable optical devices [90,91]. Additionally, polymers offer a wide range of refractive indices, which can be tailored by adjusting the chemical composition or doping. This tunability enables the design and optimization of various optical components to match specific requirements, such as dispersion compensation or efficient coupling with other optical elements. Applications of integrated optics utilizing polymer platforms include telecommunications, optical interconnects, optical sensing, biomedical imaging, and lab-on-a-chip systems. Researchers and engineers are continuously exploring new polymer materials, fabrication techniques, and device architectures to further enhance the performance and functionality of integrated optical systems [92].

**Figure 5:**
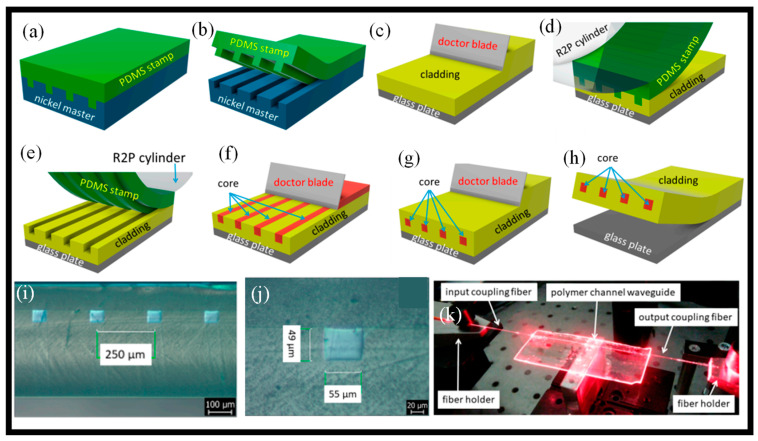
Fabrication of multimodal WG using R2P imprint (**a**–**h**) [93], (**i**) the cross-sectional view of the four optical channel WGs [93], (**j**) the detail view of the cross-section of a single optical channel WG [93], (**k**) picture of the measurement set-up with optical channel WG coupled with visible light of wavelength = 650 nm [93].

The characteristics of roll-to-plate (R2P) nanoimprinted multimode optical channel WGs made from UV-curable inorganic-organic hybrid polymers are presented [93]. The PDMS elastomer stamp was created using a nickel master mold. A photoresist master created using the photolithographic technique served as the basis for the galvanoplastic procedure that created this nickel negative mold. Sylgard 184 elastomer, which was used to make PDMS stamps, was created by combining the A and B agents in a 10:1 ratio, stirring the mixture, and then desiccating it for 60 min. The elastomer was then applied to the nickel mold, and it was allowed to be set for 20 min at 125 °C in the oven. The PDMS stamp was gently removed from the nickel mold after cooling, and it was then processed by means of a separator. The R2P machine’s cylinder was then secured with the PDMS stamp. The doctor blade approach was then used to apply the polymer Lumogen OVD Varnish 311 cladding layer with a thickness of 500 μm onto the glass substrate. Before the imprinting procedure began, the R2P machine was correctly configured. The UV light intensity, the location of the cylinder height, and the imprinting speed are the most crucial variables. The cylinder height parameters must be chosen separately for each stamp/substrate thickness together, since they are dependent on the thickness of the stamp and substrate. The R2P NIL instrument employs 395 nm LEDs as its UV light source. The PDMS stamp was imprinted onto Varnish 311 UV photopolymer once all the R2P machine’s settings had been established. Then, using a doctor blade, the UV-curable inorganic–organic hybrid polymer OrmoClear^®^FX was deposited into the U-grooves of the Varnish substrate. UV radiation at 365 nm was used for 60 s to harden the OrmoClear^®^FX core layer. The WG structure was then separated from the glass substrate, followed by the fabrication of a Varnish 311 UV photopolymer cover cladding layer using the doctor blading process as explained in Figure 5a–h. Figure 5i,j show the optical microscope image of the four-channel WGs; Figure 5i is a cross-sectional view, and Figure 5j is a detailed representation of the one-channel WG. A comprehensive image of the optical losses measuring setup is shown in Figure 5k, where it is easy to see how red light (650 nm) is connected by the optical fiber into the channel WG [93].

Polymer WG sensors have found applications in various fields, including environmental monitoring, healthcare, food safety, and industrial sensing [94]. These WGs can be used for measuring changes in the RI of a surrounding medium. By detecting the shift in the guided light’s propagation characteristics, polymer WGs enable label-free sensing of analytes in liquids or gases [17]. This can be useful for applications such as chemical sensing, environmental monitoring, or detecting biomarkers in biological samples. Polymer WGs can be functionalized with bioactive molecules such as antibodies, enzymes, or DNA probes. When target molecules bind to these bioactive coatings, it causes a change in the RI, resulting in a measurable signal [95]. Polymer WG biosensors are employed in areas such as medical diagnostics, point-of-care testing, and DNA sequencing [96]. Polymer WGs can be tailored to selectively absorb specific gases, allowing for gas-sensing applications. By monitoring changes in the light propagation caused by gas absorption, polymer WG sensors can detect and quantify the concentration of gases such as carbon dioxide (CO_2_), nitrogen dioxide (NO_2_), or volatile organic compounds (VOCs). This can be applied to indoor air quality monitoring, industrial safety, and environmental monitoring. 

Moreover, these WGs can be engineered to be sensitive to temperature or mechanical strain. Changes in temperature or strain cause alterations in the WG’s dimensions or RI, leading to detectable changes in the transmitted light [97,98]. Polymer WG sensors find applications in structural health monitoring, robotics, and aerospace industries [99]. It is important to note that the specific design and fabrication of polymer WG sensors will depend on the desired application and target sensing parameters. Different polymers, WG structures, and sensing mechanisms can be employed to optimize the sensor’s performance for a particular application.

For label-free, cost-effective biosensing and communications applications, there is a great deal of interest in polymer-based materials utilized in photonic circuits, such as benzocyclobutene (BCB), SU8, and PMMA [100,101]. Electronics and optical components are easily incorporated into polymers [102]. High-index contrast materials such as Si and Si nitride cause a significant loss in wall scattering compared to low-contrast polymer-based WGs [103,104]. This eliminates any production restrictions and makes it possible to build polymer-based WGs with a substantial footprint in a Si wafer and minimum side wall scattering loss.

Due to their integration potential and ease of manufacture using inexpensive polymer materials, μ-RRs have emerged as a crucial component for integrated optical sensors. Today, there is an increasing demand for RRs as highly sensitive and selective functions, particularly in the fields of food and health. Optical μ-RRs are the focus of several investigations as a potential label-free biosensing device for application in environmental monitoring and medical diagnosis [105]. In these RRs, the evanescent fields of the resonant light are used to examine the change in RI triggered by the presence of analytes in the surrounding medium [49]. For biosensing applications, it would be wise to utilize the premium SU-8 RRs produced by NIL [106]. The SU-8 polymer has been the topic of in-depth studies in the domains of photonics and microfluidics because of its extraordinary optical and mechanical properties, good corrosion resistance, and high thermal stability. It has a distinctive capacity to create sidewalls with straight profiles and high aspect ratios thanks to its high amount of cross-linking.

Devices that employ the optical characteristics of a polymer WG to recognize mechanical changes are known as polymer WG-based-mechanical sensors. The RI of the material fluctuates as the WG is deformed because of mechanical stress or strain, which influences how light travels through it. It is possible to quantify this alteration in the WG’s optical characteristics and utilize it to determine the existence and level of mechanical stress or strain [107]. Some possible applications for polymer WG mechanical sensors include environmental monitoring, biomedical sensing, and structural health monitoring [108,109]. Polymer WG-based mechanical sensors come in a variety of designs, such as cantilever-based sensors, μ-RR sensors, and Mach–Zehnder interferometer (MZI) sensors [17]. A polymer multimode interference (MMI) coupler-based optomechanical pressure sensor with a high sensitivity of 8.2 ppm/Pa has been developed [98]. It has been demonstrated that low humidity concentration may be detected using a polymer WG sensor with a symmetric multilayer architecture [110]. This sensor captured the resulting optical phase changes when water molecules diffused into the polymer WG. For humidity levels, it is feasible to achieve a sensitivity of several parts per million. It is also straightforward to spot trends in the index (increase or decrease) in the sensing layer, since the sensor offers the absolute indication of the shifting of the produced interference fringes. This work indicates a very bright future for the creation of a small, inexpensive disposable optical sensor for moisture-sensing applications [110].

**Figure 6:**
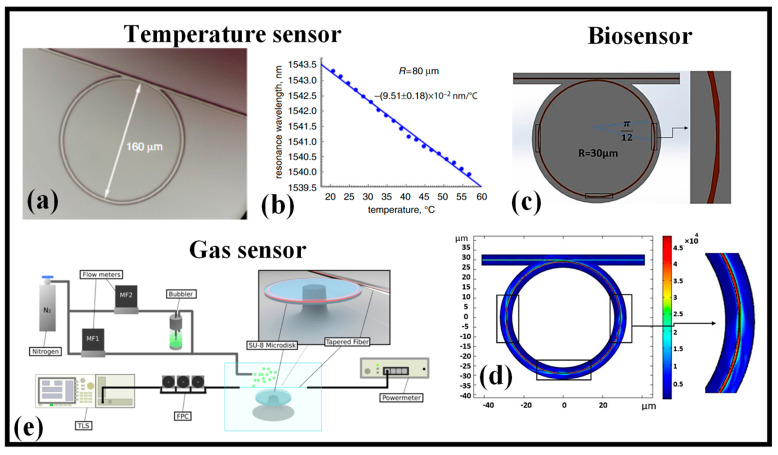
Polymer RR devices and their applications, (**a**) RR for temperature monitoring [111], (**b**) resonance wavelength of the RR versus temperature [111], (**c**) Partially tapered polymer RR [112], (**d**) E-field distribution in the RR at resonance wavelength [112], (**e**) Schematic of the experimental setup used in the gas sensing experiment [117].

It is demonstrated that a μ-RR can sense temperature and RI by observing the shift in the resonance wavelength of the interferometric pattern [111]. This μ-RR was created by means of femtosecond laser two-photon photopolymerization (TPP) in an epoxy-based negative photoresist (SU-8). The experimental results show sensitivity to temperature of 9.51 × 10^−2^ nm/°C (blueshift) and sensitivity to the RI of 12.50 nm/RIU (redshift), respectively. Following spin-coating on a glass substrate, a thin-film of photoresist SU-8 with a RI of 1.575 at 1550 nm is then baked. An objective lens is then utilized to focus an 800 nm Ti:sapphire femtosecond laser with a pulse width of 67 fs and pulse energy of 1.6 nJ on the SU-8 film. The ring and bus WG are etched into the SU-8 film using TPP by directing the movement of the X, Y, and Z translation stages that have been computer-programmed. On the glass slide, a gapless μ-RR with a radius of 80 μm is created after baking and developing, as illustrated in Figure 6a [111]. The resonance wavelength performs a blueshift as the RI of SU-8 decreases due to the increase in the ambient temperature as shown in Figure 6b [111]. 

An investigation was performed into the properties of a polymer RR with a partly tapered WG for biomedical sensing, as shown in Figure 6c [112]. The E-field distribution in the RR at resonance wavelength is shown in Figure 6d. The objective was to create a biosensor with a better figure of merit (FOM) that is more sensitive. The idea was to increase field contact, with the sample being tested in tapered segments. Here, the WG width is gradually cut in half. Sensitivity increases from 84.6 nm/RIU to 101.74 nm/RIU with a relatively slight Q-factor drop from 4.60 × 10^3^ for a strip WG to 4.36 × 10^3^ for a partly tapered one. Following the study, the calculated FOM increases from 497 for a strip ring to 565 for a π/4 tapered ring close to six tapered ones, with the number of tapered parts ranging from zero to fifteen. It is recommended to use the three-tapered one while evaluating the manufacturing method. The device is made entirely of polymer and offers the benefits of a disposable, low-cost biosensor that is compatible with roll-to-roll production. This approach may also be used with polymer- or Si-based devices with an isolator to benefit from a higher Q-factor and improved sensitivity [112].

In many modern applications, including medical diagnosis and air quality monitoring, gas sensors are employed extensively. They have been created using mechanical, optical, thermal, and electrochemical detecting approaches. Despite the recent focus on electrochemical gas sensors [113], optical gas sensors are seen as a better choice because they can be utilized in explosive media and are appropriate in hostile environments [114]. They are also immune to EM noise. An optical cavity known as a whispering gallery mode (WGM) resonator has been extensively studied for gas sensing as well as for the detection of bacteria, proteins, and nanoparticles [115,116]. Total internal reflection in these resonators confines light to a cavity with a rounded shape, forcing it to circle the object. Their high quality factor (Q) and low modal volume are widely recognized. Polymeric microdisks’ sensitivity is assessed for several substances in their vapor phase, including humidity, isopropanol, toluene, limonene, 1-butanol, and pentanoic acid (valeric acid) [117]. Pentanoic acid has the greatest sensitivity (23 pm/ppm) and an estimated limit of detection of 0.6 ppm among these chemicals. To enhance the functionality of gas sensing devices, it may be possible to design the geometric distortion caused by polymer swelling. The ratio of the undercut over the microcavity’s radius increases with humidity sensitivity, according to experimental studies.

Fast readout of the transmission spectrum from the WGM microcavity while submerged in an analyte concentration is possible using the experimental setup shown in Figure 6e. A tunable IR laser source linked to a 1 μm tapered fiber optically coupled to the cavity is used to test the optical transmission of the cavity. To modify the coupling between the tapered fiber and the microcavity, a sample is mounted on one five-axis stage and the tapered fiber on another. To increase the optical signal’s ER, a fiber polarization controller is employed. An optical power meter is used to determine the output signal as the tunable laser’s wavelength changes to create a spectrum [117].

### 4.3. Plasmonic Platform

Plasmonic-based devices have attracted a lot of attention in recent years because of their distinctive characteristics, which make it possible to significantly increase the sensitivity of photonic sensors [23,118]. The role of metals in the creation of novel optical devices based on plasmonic phenomena such as SPPs is changing because of ongoing advances in nanofabrication [119]. These have a wide range of applications that are appealing, including nanophotonics, biosensing, electronics, imaging, and many more [120,121,122]. SPPs are tightly bonded to the metal–dielectric contacts, penetrating as far down as 10 nm into the metal (skin depth) and frequently as far as 100 nm or more in the dielectric material. 

Gold (Au) is one of the most extensively studied and utilized plasmonic metals. It has excellent plasmonic properties in the visible to near-infrared range, making it suitable for various applications, including biosensing, imaging, and nanophotonics. Au nanoparticles, in particular, are extensively employed due to their tunable plasmonic resonance and stability. Silver (Ag) is another popular plasmonic metal that exhibits strong plasmonic behavior across a broad spectral range, including the visible, near-infrared, and ultraviolet regions. It possesses high conductivity and a high degree of light confinement, making it useful for applications such as surface-enhanced Raman spectroscopy (SERS), sensing, and optical nanocircuits. Copper (Cu) is gaining attention as a plasmonic material due to its low cost and compatibility with CMOS technology. Although Cu has higher optical losses compared to Au and Ag, it still exhibits plasmonic behavior in the near-infrared range, making it suitable for applications such as telecommunications and energy harvesting. Plasmonic metals owe their unique properties to the collective behavior of electrons in the metal nanoparticles, which can interact strongly with light at the nanoscale. These materials play a crucial role in the field of nanophotonics and have numerous applications in various areas of science and technology.

Among plasmonic-based nanostructures used in integrated photonic circuits, the metal–insulator–metal (MIM) WG system is one of the most prevalent. In comparison to sensing devices based on other platforms, for instance, Si photonics or optical fiber, plasmonic sensors are very desirable and in high demand due to their compact size and strong sensing characteristics. Due to their capacity to surpass the diffraction limit of light, SPP WG structures, specifically MIM WGs, have received considerable consideration [123]. Due to their small size, ease of integration, and favorable balance between light localization and transmission loss, MIM WGs are expected to enable the realization of highly integrated optical circuits [124,125].

An MIM WG-based sensor is a type of optical sensor that uses a WG consisting of an MIM structure to detect variations in the RI or the presence of biomolecules on its surface [126]. The MIM WG typically consists of a thin insulator layer (air) sandwiched between two metal layers. SPPs are EM waves that propagate along the interface between a metal and a dielectric material, such as air or a biological medium [127]. They arise from the coupling between the free electrons in the metal and the EM field of the light. SPPs have unique properties that make them attractive for applications in various fields such as sensing, imaging, and communication [22]. One of the most important properties of SPPs is their ability to confine EM energy to subwavelength dimensions, which can enhance the sensitivity of sensing and imaging devices. SPPs can also be used to manipulate light at the nanoscale, enabling the development of new photonic devices with unprecedented functionality. The study of SPPs has become a very active area of research in recent years, with many researchers exploring new ways to manipulate and control these waves for practical applications [126,128].

Plasmonic resonator cavities are formed by creating a nanoscale cavity side coupled to an MIM bus WG [129,130]. When light propagating in the MIM WG satisfies the resonance condition of the cavity, it excites plasmons in the metal, which can be trapped and resonantly coupled to the cavity mode. The resonance wavelength of the cavity is very sensitive to changes in the RI of the surrounding medium, which allows it to be used as a sensor [131]. Plasmonic sensors based on resonator cavities have potential applications in areas such as chemical and biological sensing, environmental monitoring, and medical diagnostics. They offer advantages such as high sensitivity, label-free detection, and the ability to perform real-time measurements. However, there are also challenges associated with their design and fabrication, such as achieving high-Q resonances, optimizing the coupling between the plasmons and the cavity mode, and minimizing background noise.

**Figure 7:**
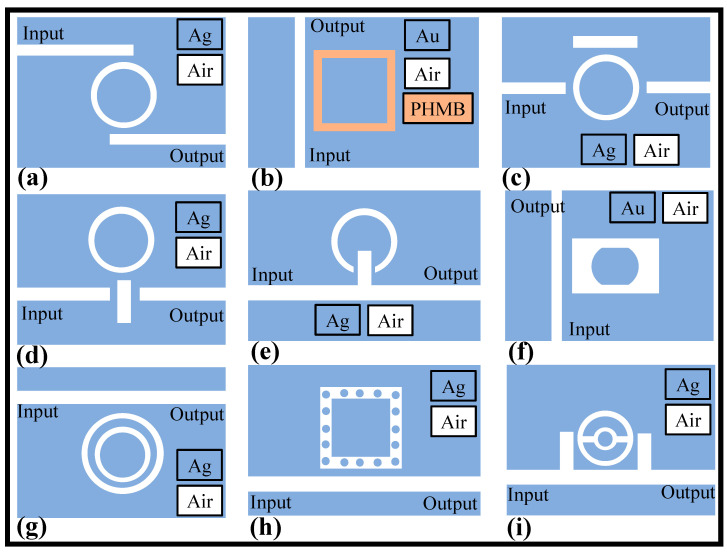
Schematic of some of the few recently proposed plasmonic RR-based sensors with unique cavity shapes, (**a**) ring resonator [136], (**b**) square ring cavity filled with a functional polymer [29], (**c**) ring MIM WG side coupled with a rectangular resonator [138], (**d**) rectangular resonator and a ring resonator [139], (**e**) notched ring resonator [140], (**f**) rectangular hollow cavity with metallic island [137], (**g**) concentric double MIM rings [141], (**h**) square ring-shaped resonator containing silver nanorods [142], (**i**) connected concentric double ring resonator [143].

One of the advantages of MIM WG-based RRs is that they can achieve a very high-FOM which is calculated as sensitivity/FWHM, where FWHM is the full width at half the maximum of the resonant wavelength. Fano resonance has a noticeably asymmetric line form and results from the coherent coupling and interference between a discrete state and a continuous state. The Fano resonance has demonstrated significant potential in the sensor, switching, nonlinear, and slow light sectors due to the ease with which high sensitivity and large FOM may be obtained. The FOM of the Fano-based plasmonic sensors reach as high as 10^5^ [132]. MIM WG-based RRs can be fabricated using electron beam lithography and deposition techniques, making them compatible with other photonic components on a chip. They can also be integrated with other materials, such as graphene or quantum dots, to enhance their performance for specific applications [133]. In recent times, several plasmonic RR devices have been proposed with diverse shapes of the cavities for RI, temperature, and gas sensing applications [3,121,123,134,135,136,137]. The appropriate shape and size of the cavity help in enhancing the sensitivity of the device. Figure 7a–i present MIM WG-based RRs with unique cavity shapes for sensing applications. 

In [144], a plasmonic sensor for measuring RI is described that incorporates a ring resonator with circular tapered defects connected to an MIM WG with tapered defects. The device’s sensitivity is around 1295 nm/RIU, but because of the complexity of its design, even a manufacturing mistake of a few nanometers can impair the device’s performance. Another complex RI sensor with two symmetric triangular stubs on an MIM WG connected to a cavity with a circular split-ring resonator is presented [135]. The device has a sensitivity of 1500 nm/RIU. To obtain the best sensing performance with this sensor arrangement, numerous parameters must be carefully tuned. The designs suggested in [134,145] experience a similar circumstance. The numerical findings shown in this research appear to be appealing, but the actual difficulty arises at the manufacturing stage of these devices, where several factors need to be tuned at a nanoscale level.

An essential type of sensor used to monitor temperature in several applications is the temperature sensor. Optical temperature sensors have more benefits than traditional electrical temperature sensors, including immunity to EM interference, high sensitivity, a wide temperature range, quick response, and stability. Recent research has shown that MIM plasmonic WG devices may be effectively used for temperature monitoring applications when linked with thermal sensing mediums such as ethanol or PDMS [146,147,148]. Zhu et al. developed a sensor device with a very high sensitivity of −3.64 nm/°C that can only be utilized for temperature sensing [149]. Additionally, Zhu et al. used a polydimethylsiloxane (PDMS)-sealed semi-square ring resonator to numerically investigate a small Fano resonance temperature sensor [146]. A temperature sensor using ethanol in a resonant cavity with 0.36 nm/°C sensitivity was proposed by Kong et al. [150]. 

**Figure 8:**
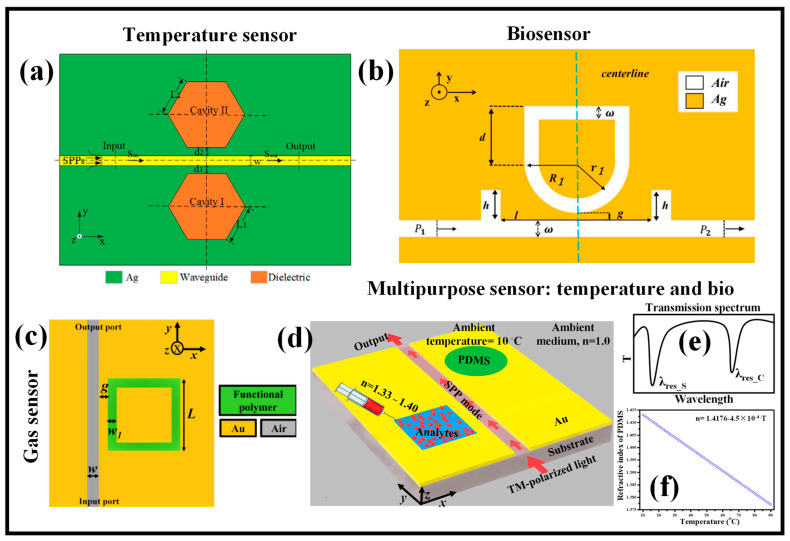
MIM WG-based (**a**) temperature sensor [151], (**b**) biosensor [152], (**c**) CO_2_ gas sensor [29], (**d**) simultaneous temperature sensor and biosensor [157], (**e**) transmission spectrum [157], (**f**) dependence of refractive index of PDMS on ambient temperature [157].

The finite-difference time-domain (FDTD) approach is used to numerically examine a plasmonic temperature-sensing device that is built on an MIM WG with dual side-coupled hexagonal cavities as shown in Figure 8a [151]. According to the outcomes of the numerical simulation, a resonance dip may be seen in the transmission spectrum. Additionally, by adjusting the coupling distance between the WG and two cavities, the FWHM of the resonance dip can be reduced and the extinction ratio (ER) can reach a maximum value. The resonance dip and ambient temperature have a linear connection, which is the basis for the discussion of the temperature-sensing properties. The side length and coupling distance affect the temperature sensitivity. To explore the temperature-sensing resolution based on spectral interrogation, two concepts—optical spectrum interference (OSI) and misjudge rate (MR)—are also presented for the first time [151]. This technique is important for the development of high-temperature sensitivity and high-sensing resolution nanoscale optical sensors.

A unique MIM WG with two rectangular baffles and a U-shaped ring resonator (USRR) were combined to create the RI sensor construction as shown in Figure 8b [152]. The transmission characteristics of the sensor were theoretically inspected using the finite element method (FEM). The simulation findings demonstrate that the discrete narrow-band mode and the succeeding wide-band mode combine to produce the acute asymmetric resonance known as the Fano resonance. The development of the broadband and narrowband is then further investigated, and eventually, the main variables impacting the sensor’s performance are discovered. The FOM for this refractive-index sensor is 53.16, and its optimum sensitivity is 2020 nm/RIU [152]. 

In [153], a functional polymer-coated metasurface perfect absorber made of polyhexamethylene biguanide (PHMB) is presented for use in CO_2_ monitoring. This absorber can detect gases with concentrations ranging from 0 to 524 ppm. A CO_2_ concentration of 434 ppm resulted in a maximum sensitivity of 17.3 pm/ppm. A Si ring resonator CO_2_ gas sensor was shown in [154] with a functional PHMB layer placed over the WG structure. This approach has a detection limit of 20 ppm and can detect gas concentrations up to 500 ppm. Additionally, a Si dual gas sensor built on a wavelength-multiplexed ring resonator array was suggested in [155] for the real-time detection of H_2_ and CO_2_ gases. Additionally, an experimental study of a Fabry–Perot interferometric optical fiber sensor for CO_2_ gas detection was conducted. For a gas range of 0–700 ppm, the suggested sensor offers a sensitivity of 12.2 pm/ppm [156].

However, to boost the sensitivity of the device, plasmonic MIM WG-based sensors are preferred. The numerical investigation of a simple and highly sensitive CO_2_ gas sensor device is presented in [29]. The sensor is composed of a square ring cavity containing a PHMB functional material and a plasmonic MIM WG side, as shown in Figure 8c. When exposed to CO_2_, the functional material’s RI changes, and this change is linearly proportional to the gas’s concentration. The SPP wave-based sensors are extremely sensitive because the EM wave interacts strongly with the substance. Plasmonic sensors offer a platform with a maximum sensitivity of ~135.95 pm/ppm, which is not possible with optical sensors built on Si photonics, achieved by deploying the PHMB polymer in the MIM WG.

For accurate findings, the testing of biological samples at diagnostic centers needs to be performed in a temperature-controlled setting. Therefore, a lab-on-chip solution is needed that can examine the analytes and temperature simultaneously. An MIM WG-based multifunctional plasmonic sensor device was suggested by Kazanskiy et al., as shown in Figure 8d [157]. Temperature sensing and biological sensing applications may both immediately use the sensor architecture. The sensor is composed of two regular resonant cavities that are square and circular, with one side connected to an MIM bus WG. The analytes for the biosensing task can be infused into the square cavity, and a thermo-optic polymer can be placed in the circular cavity to provide a change in resonance wavelength in response to changes in ambient temperature. The two sensor systems operate separately. Each cavity offers a resonance dip at a particular point in the sensor’s transmission range that does not obstruct analysis, as shown in Figure 8e. The correlation between the RI of the PDMS layer and the ambient temperature is plotted in Figure 8f. It can be seen that the RI of the polymer reduces as the ambient temperature increases. A basic setup incorporated on a single chip can give a sensitivity of 700 nm/RIU and 0.35 nm/°C for biosensing and temperature sensing, respectively. In addition, the FOM for the biosensing module and temperature sensing component is ~21.9 and 0.008, respectively. Table 2 reports some of the recently proposed MIM WG-based plasmonic sensing devices for temperature, gas and biosensing applications. 

## 5. Limiting Factors of RR Devices

RRs have several limitations that can affect their performance. For instance, one of the major limitations of RRs is propagation losses due to the ring’s finite size, which can lead to increased attenuation of the light signal passing through the device [165]. The losses increase with the number of times the light must travel around the ring, which can limit the overall performance of the device [166]. The free spectral range (FSR) is the frequency range over which the resonator can support resonant modes. The FSR of an RR is determined by the size of the ring and the RI of the material, and it can be a limiting factor in the design of the device [167]. Fabrication tolerances can also have a significant impact on the performance of RRs, especially when the devices are miniaturized. Small changes in the ring’s diameter or other geometrical parameters can affect the device’s resonance frequency and bandwidth, which can lead to reduced performance [49]. The resonance frequency of RRs can shift with temperature changes, which can be a limiting factor in their use [168]. Thermal drift can be caused by changes in the RI of the material, changes in the ring’s dimensions due to thermal expansion, or changes in the effective RI of the WG due to thermal effects. Moreover, RRs are usually managed in arrays to construct more complex optical devices. In these applications, crosstalk between adjacent RRs can limit the performance of the overall device. Crosstalk can be caused by coupling between the rings or by interference between the resonant modes of neighboring RRs [169].

## 6. Concluding Remarks

Optical RR sensors offer several advantages that make them attractive for various applications. This photonic structure exhibits high sensitivity to changes in the RI of the adjacent medium. This makes them superlative for sensing applications where small changes in the environment need to be detected. Optical RRs can detect analytes without the need for any labels or markers. This simplifies the sensing process and eliminates the need for complex sample preparation steps. These structures can be fabricated on small, chip-scale platforms, allowing for miniaturization and integration into compact sensor devices. Due to their small size and the confinement of light within the resonator, optical RR sensors can provide rapid response times. This makes them suitable for real-time monitoring and dynamic sensing applications. 

RRs can be designed to operate at different wavelengths, allowing for the recognition of a wide range of analytes or physical parameters. This versatility makes them applicable in various sensing scenarios. Optical RRs can be utilized in different sensing modes, including absorption, RI, and surface-enhanced sensing. This flexibility enables their use in diverse sensing applications across different fields. Multiple RRs can be integrated on a single chip, permitting the simultaneous sensing of multiple analytes or parameters. This multiplexing capability boosts the efficiency and throughput of the sensing system. Optical RR sensors typically operate at low power levels, making them energy-efficient and suitable for battery-powered or remote sensing applications. Generally, sensors based on RR structures offer a combination of high sensitivity, compact size, fast response, and versatility, making them valuable tools for a wide range of sensing applications in areas such as environmental monitoring, biomedical diagnostics, and chemical analysis.

Si photonics allows for the integration of various photonic components, such as modulators, detectors, and WGs, onto a single chip alongside electronic component. This high level of integration enables the development of compact and complex photonic circuits, which can lead to smaller and more efficient devices. This high level of integration enables compact and complex photonic circuits, which can lead to smaller and more efficient devices. The use of Si as a substrate material allows for large-scale, cost-effective production using existing manufacturing facilities. This scalability and cost-effectiveness make Si photonics attractive for mass production and commercialization, enabling widespread adoption across various industries. Si photonics has the potential for integration with other emerging technologies, such as quantum computing and artificial intelligence. By combining photonics with these fields, new applications and functionalities can be realized, further expanding the capabilities of Si photonics-based systems.

In general, polymer materials are less costly than conventional WG materials such as Si, glass, or silica. This increases the cost-effectiveness of polymer RRs for mass manufacture, enabling their usage in high-volume applications. Polymer WGs can be fabricated using relatively simple and cost-effective manufacturing techniques, such as spin coating, hot embossing, nanoimprint lithography, molding, or direct laser writing. These techniques allow for rapid prototyping and high-throughput production, reducing the overall fabrication time.

Plasmonic devices can operate at subwavelength scales, enabling the miniaturization of components and integration with nanoscale devices. This capability is crucial for applications such as on-chip optical communications, nanophotonics, and biosensing, where compactness and high device density are desired. Plasmonic devices can operate at subwavelength scales, enabling the miniaturization of components and integration with nanoscale devices. This capability is imperative for applications such as on-chip optical communications, nanophotonics, and biosensing, where compactness and high device density are desired. Therefore, RR structures realized on the plasmonic platform are highly sensitive to any minor change in the ambient medium as a result their sensitivity is significantly higher than the one offered by RRs developed on semiconductor and polymer platforms. Moreover, the footprint of plasmonic WGs is unmatchable by the other optical platforms. 

These advantages make plasmonics a promising platform for various applications, including optical communications, sensing, imaging, computing, and energy harvesting. However, it is important to note that plasmonics also faces challenges, such as high losses and fabrication complexities, which need to be addressed for widespread practical implementation.

## Figures and Tables

**Table 1:** Recently reported RRs based on semiconductor platform for temperature, gas, and biosensing applications.

WG Type	Application	Sensitivity	Q-Factor	Numerical/Experiment	Ref.
Ridge WG	Bio	380 nm/RIU	10,000	Experiment	[78]
SWG hybrid plasmonic WG	Bio	1000 nm/RIU	2569.8	Numerical	[79]
Metal-assisted ridge WG	Bio	300 nm/RIU	201.6	Numerical	[80]
Slotted RR	Gas	490 nm/RIU	5000	Experimental	[75]
Ridge	Temperature	293.9 pm/°C	-	Experimental	[67]
1D-PhC RR	Bio	248 nm/RIU	-	Experimental	[72]
Ridge	Temperature	83 pm/°C	-	Experimental	[81]
Freestanding RR	Gas	1.7 pm/1000 ppm	30,000	Experimental	[82]
Hybrid microcavity	Bio	497 nm/RIU	600	Numerical	[83]
Double slot RR	Bio	433.33 nm/RIU	4325	Numerical	[84]

**Table 2:** Recently reported MIM WG-based plasmonic sensors for temperature, gas, and biosensing applications.

Cavity Shape	Material	Application	Sensitivity	Wavelength Range (nm)	FOM	Ref.
Hexagonal	Ag	Temperature	0.45 nm/°C	1400–1750	0.013	[151]
Square	Au	Gas	135.95 pm/ppm	900–1500	-	[29]
Square cross	Au	Bio	825.7 nm/RIU	950–1450	13.14	[158]
Concentric double rings	Ag	Bio and temperature	2260 nm/RIU and 1.48 nm/°C	2300–3800	56.5	[143]
Square split ring	Ag	Bio	1290.2 nm/RIU	500–1400	3.6 × 10^4^	[159]
Two stub and one slot resonator	Ag	Gas	124 pm/ppm	800–1900	-	[160]
Side-coupled and ring-encapsulated circular	Au	Temperature and bio	−0.58 nm/°C and −0.64 nm/°C; 1240 nm/RIU and 1350 nm/RIU	1500–2000	8.6 and 1955.2 (for temperature); 18.74 and 691 (for bio)	[161]
Defective oval	Ag	Temperature	2.463 nm/°C	800–3400	2.27 × 10^4^	[162]
Elliptical	Ag	Bio	550 nm/RIU	500–1700	282.5	[163]
Semi-ring	Ag	Bio	1260.5 nm/RIU	600–1800	41.67	[164]

## Data Availability

Not applicable.

## Abbreviations

Waveguide = WG; Ring resonator = RR; Whispering gallery mode = WGM; Refractive index = RI; Surface plasmon resonance = SPR; Surface plasmon polariton = SPP; Free spectral range = FSR; Metal-insulator-metal = MIM; Figure of merit = FOM; Full width at half maximum = FWHM; Polyhexamethylene biguanide = PHMB; Finite element method = FEM; Finite difference time domain = FDTD; Electron beam lithography = EBL; Nanoimprint lithography = NIL; Silicon-on-insulator = SOI; Subwavelength grating = SWG; Photonic crystal = PhC; Complementary metal-oxide-semiconductor = CMOS; Roll-to-plate = R2P.
